# 

**Published:** 1893-03-11

**Authors:** 


### CONTENTS.

The Marquis of Salisbury at Oxford . . . . . 371

Around the Hospitals . . . . . . . . . 372

Medical History from the Earliest Times. By E. T. Withington, M.A., M.B. Oxon. XLVI.—The Mystics, Sympathy, and Signatures . . . . . . . . . 373

Pensions for Old Age. I.—Three Schemes Considered . 374

Diet in Disease. By Mrs. Ernest Hart. XV.—Diabetes (*con.*) 375

Everybody's Page . . . . . . . . . 376

Annotations.—Catholicism and Physiology—The Enthusiasm of Medicine—More Cholera Precautions . . . . . . 377

Notes and News . . . . . . . . . 378

Hospital Opinion.—Overstrained Brains; a Cry for More Institutions . . . . . . . . . 386

Scraps and Gleanings . . . . . . . . . 386

The Hospital Clinic—

Guy's Hospital.—The Treatment of Fractures of the Femur . 379

Royal Infirmary, Edinburgh.—Treatment of the Bowel in Operations for Strangulated Hernia . . . . . . 380

The Royal Southern Hospital, Liverpool.—Treatment of Pleurisy . . . . . . . . . 381

The Practitioners' Bookshelf.—"Quain's Elements of Anatomy" . . . . . . . . . 382

The Institutional Workshop—Within the Hospitals.—The Children's Hospital, Great Ormond Street . . . . . 383

Hospitals of the World. I.—Early Hospitals in Europe . 385

Church of England Sanitary Association . . . . 386

The Book World of Medicine and Science.

The Student of Medicine.

### EXTRA SUPPLEMENT.—THE NURSING MIRROR.

News from the Nursing World.—Princess Henry of Battenberg—Trained Nurses' Annuity Fund—Too Late—Sense without Syrup—Convalescent from Fever—A New Hospital—Nursing and Nursed—Outdoor Relief—Chipping Nurses' Home—At Home by the Sea—Dr. James Anderson—Hull Royal Infirmary—Reasonable Regulations—Nurses for India—Short Items—The Disabled Nurse—"The Hospital" Endowed Bed . . . . . clxxiii

Management of Consumption . . . . . . . clxxv

The International Congress of Charities, Correction, and Philanthropy . . . . . . . clxxv

Asylum Nursing . . . . . . . . . clxxvi

Royal Edinburgh Asylum, Morningside . . . clxxviii

Everybody's Opinion.—Soothing Syrups—The Young Nurse on Herself—American Experiences—Nurses in Canada—Welcome Gift—Co-operation for Nurses—More Co-operation—Carbolised Sheet—"The Hospital" Endowed Bed—Infection in Holland . . . . . . clxxvii

Notes and Queries . . . . . . . . . clxxviii

For Reading to the Sick.—The Yoke . . . . . clxxix

Our Patient.—Where to Go . . . . . . . clxxix

The International Nursing Congress.—Mrs. Bedford Fenwick's Position . . . . . . . . . clxxix

City Asylum, Birmingham.—Minor Appointments . clxxix

The Muses' Looking Glass.—Wants and Workers . clxxx

The Book World for Women and Nurses.

